# Validation of an *International Classification of Disease, Ninth Revision* coding algorithm to identify decompressive craniectomy for stroke

**DOI:** 10.1186/s12883-017-0864-8

**Published:** 2017-06-26

**Authors:** Hormuzdiyar H. Dasenbrock, David J. Cote, Yuri Pompeu, Viren S. Vasudeva, Timothy R. Smith, William B. Gormley

**Affiliations:** Cushing Neurological Outcomes Center, Brigham and Women’s Hospital, Harvard Medical School, 75 Francis Street, Boston, MA 02115 USA

**Keywords:** Decompressive craniectomy, International classification of disease, Ischemic stroke, Validation

## Abstract

**Background:**

Although *International Classification of Disease, Ninth Revision, Clinical Modification* (ICD9-CM) coding is the basis of administrative claims data, no study has validated an ICD9-CM algorithm to identify patients undergoing decompressive craniectomy for space-occupying supratentorial infarction.

**Methods:**

Patients who underwent decompressive craniectomy for stroke at our institution were retrospectively identified and their associated ICD9-CM codes were extracted from billing data. An ICD9-CM algorithm was generated and its accuracy compared against physician review.

**Results:**

A total of 10,925 neurosurgical operations were performed from December 2008 to March 2015, of which 46 (0.4%) were decompressive craniectomy for space-occupying stroke. The ICD9-CM procedure code for craniectomy (01.25) was only encoded in 67.4% of patients, while craniotomy (01.24) was used in 19.6% and lobectomy (01.39, 01.53, 01.59) in 13.1%. The ICD-9-CM algorithm included patients with a diagnosis codes for cerebral infarction (433.11, 434.01, 434.11, and 434.91) and a procedure code for craniotomy, craniectomy, or lobectomy. Patients were excluded with an ICD9-CM diagnosis code for brain tumor, intracranial abscess, subarachnoid hemorrhage, vertebrobasilar infarction, intracranial aneurysm, Moyamoya disease, intracranial venous sinus thrombosis, vertebral artery dissection, congenital cerebrovascular anomaly, head trauma or an ICD9-CM procedure code for laminectomy. This algorithm had a sensitivity of 97.8%, specificity of 99.9%, positive predictive value of 88.2%, and negative predictive value of 99.9%. The majority of false-positive results were patients who underwent evacuation of a primary intracerebral hematoma.

**Conclusion:**

An ICD-9-CM algorithm based on diagnosis and procedure codes can effectively identify patients undergoing decompressive craniectomy for supratentorial stroke.

## Background

*International Classification of Diseases, Ninth Edition, Clinical Modification* (ICD9-CM) coding has been increasingly used in medical research. Administrative claims data, including Medicare claims files and hospital administrative records, comprise data that are collected for billing: such data have also been employed for research, and are particularly useful for rare diseases or procedures, where the number of patients at a single center may be comparatively small [1]. Additionally, administrative data have been used to evaluate temporal trends in management of conditions [2]. However, the validity of administrative claims research is dependent upon the accuracy of the ICD9-CM coding upon which it is based.

Although several studies have evaluated the accuracy of ICD9 based coding to identify acute ischemic stroke [3–13], patients with space-occupying supratentorial cerebral infarction represent a unique subset of stroke, in whom decompressive hemicraniectomy may be mortality and morbidity sparing [14–16]. Nevertheless, no ICD9 based algorithm has specifically examined patients undergoing decompressive craniectomy for stroke. The goal of this analysis was to construct and validate against physician review an ICD9-CM based algorithm to identify patients undergoing decompressive craniectomy for stroke.

## Construction and content

All decompressive craniectomies performed for acute space-occupying cerebral infarction between December 15, 2008 and March 15, 2014 were retrospectively identified by physician review after approval by our institutional review board. Thereafter, our institutional billing data were queried to evaluate the concordance of designated coding with physician chart review. The primary documented ICD9-CM procedure code and all ICD9-CM diagnosis codes for patients who underwent a neurosurgical intervention during the selected time period were analyzed. Thereafter, ICD9-CM diagnosis and procedure codes that were pertinent to decompressive craniectomy for supratentorial space-occupying infarction were identified. These codes were then used to query all patients who underwent a neurosurgical intervention. The results of this initial query were used to identify pertinent ICD9-CM exclusion criteria, based on patients retrieved by the initial criteria but who had not undergone craniectomy for supratentorial cerebral infarction. Finally, the ICD9-CM based algorithm involving both inclusion and exclusion criteria was constructed, and it was applied to billing data from our institution during this time period. The final algorithm was selected as the combination of inclusion and exclusion criteria that optimized the classification of patients who underwent decompressive craniectomy.

Additionally, the ability of ICD9-CM coding to indicate hemorrhagic conversion of infarction was evaluated. Hemorrhagic conversion was defined as any intraparenchymal hemorrhage noted on review of the radiology reports of the patient’s postoperative computed tomography or magnetic resonance imaging. Postoperative extra-axial and petechial intraparenchymal hemorrhages were not considered to be hemorrhagic conversion.

### Statistical analysis

Descriptive statistics were performed evaluating patients who underwent decompressive craniectomy for infarction. Thereafter, the sensitivity, specificity, positive and negative predictive values of the ICD9-CM based algorithm were determined by comparing patients identified with this approach to those known to have undergone decompressive craniectomy for space-occupying infarction, as determined by physician review of the operative note. All data analysis was performed with IBM® Statistical Package for the Social Sciences (SPSS)® version 23 (IBM Inc., 2014). 

## Utility

Between December 15, 2008 and March 15, 2014, a total of 10,925 operations were performed by a neurosurgeon at our institution, of which 46 (0.4%) were decompressive craniectomy for acute space-occupying infarction. The mean age of patients was 54.3 (standard deviation: 10.7, range: 23–72) years, 65.2% (*n* = 30) were male and 34.8% (*n* = 16) were female. The laterality of the infarction was right-sided on 69.6% (*n* = 32) and left-sided on 30.4% (*n* = 14). The operation performed was a decompressive craniectomy with duraplasty in 82.6% (*n* = 38) of patients; however, 17.4% (*n* = 8) underwent decompressive craniectomy and duraplasty with concomitant excision of infarcted region (anterior temporal lobectomy alone in 7, with temporal and frontal lobectomy in 1). The ICD9-CM code indicating cerebral infarction was 433.11 (occlusion and stenosis of the carotid artery with infarction) in 26.1% (*n* = 12) of patients, 434.01 (cerebral thrombosis, with infarction) in 2.2% (*n* = 1), 434.11 (cerebral embolism, with infarction) in 41.3% (*n* = 19), and 434.91 (cerebral artery occlusion, unspecified, with infarction) in 28.3% (*n* = 13). One patient (2.2%) had an inaccurate ICD9-CM diagnosis code of 433.01 (occlusion and stenosis of the basilar artery with infarction). The ICD9-CM procedure code recorded was craniectomy (01.25) in 67.4% (*n* = 31), craniotomy (01.24) in 19.6% (*n* = 9), other incision of the brain (01.39) in 2.2% (*n* = 1), lobectomy of the brain (01.53) in 8.7% (*n* = 4), and other excision of lesion of the brain (01.59) in 2.2% (*n* = 1).

An ICD9-CM based algorithm was constructed to identify patients undergoing decompressive craniectomy for stroke (Table 1). Application of this algorithm to our institutional billing data for all patients who underwent a neurosurgical operation during the study period identified 51 patients, of whom 45 underwent decompressive craniectomy for stroke and 6 were false positives. The majority of false positive patients underwent evacuation of an intracerebral hematoma (Table 2). During the same period, 33 patients underwent posterior fossa craniectomy for infarction, all of whom were excluded with this algorithm. Only one patient who underwent decompressive craniectomy for a supratentorial stroke was not identified using this algorithm (and thereby a false negative), in whom the cerebral infarction was miscoded as occlusion and stenosis of the basilar artery with infarction. Therefore, the ICD9-CM based algorithm had a sensitivity of 97.8%, specificity of 99.9%, positive predictive value of 88.2%, and negative predictive value of 99.9%.

**Table 1:** ICD9-CM based algorithm for identifying decompressive craniectomy for stroke

Classification	Code	Definition
Inclusion Criteria		
ICD9-CM Diagnosis Codes	433.11	Occlusion & stenosis of the carotid artery, with infarction
	434.01	Cerebral thrombosis, with infarction
	434.11	Cerebral embolism, with infarction
	434.91	Cerebral artery occlusion, unspecified, with infarction
ICD9-CM Procedure Codes	01.24	Craniotomy
	01.25	Craniectomy
	01.39	Other incision of brain
	01.53	Lobectomy of brain
	01.59	Other excision of lesion or tissue of the brain
Exclusion Criteria		
ICD9-CM Diagnosis Codes	191.x	Malignant neoplasm of the brain
	198.3	Secondary malignant neoplasm of the brain and spinal cord
	324.0	Intracranial abscess
	430	Subarachnoid hemorrhage
	433.01	Occlusion & stenosis of the basilar artery, with infarction
	433.21	Occlusion & stenosis of the vertebral artery, with infarction
	437.3	Cerebral aneurysm, unruptured
	437.5	Moyamoya disease
	437.6	Intracranial venous sinus thrombosis
	443.24	Dissection of the vertebral artery
	747.81	Congenital anomalies of the cerebrovascular system
	800.xx	Fracture of the vault of the skull
	801.xx	Fracture of the base of the skull
	851.xx	Traumatic cerebral contusion
	852.xx	Traumatic subarachnoid, subdural, or extradural hemorrhage
	853.xx	Other and unspecified intracranial hemorrhage after injury
	854.xx	Intracranial injury of other and unspecified nature
ICD9-CM Procedure Codes	03.09	Laminectomy

Abbreviations: *ICD9-CM* international classification of diseases, ninth revision, clinical modification

**Table 2:** Details of the false positive patients determined by the ICD9-CM algorithm

Number	Diagnosis	Operation	ICD9-CM Procedure Code	ICD9-CM Diagnosis Code
1	Left ICH after TPA administration for left MCA stroke	Left decompressive craniectomy and evacuation of hematoma	01.24	434.91
2	Right temporal ICH	Right frontotemporal craniotomy for evacuation of hematoma	01.39	434.11
3	Right frontoparietal ICH	Right decompressive craniectomy and evacuation of hematoma, left frontal ventriculostomy	01.24	434.11
4	R frontal ICH	Right frontal craniotomy for evacuation of hematoma	01.39	434.91
5	Left parietal ICH secondary to disseminated aspergillosis with refractory intracranial hypertension	Left decompressive craniectomy	01.25	434.11
6	Right frontal ICH	Right frontotemporal craniotomy for evacuation of hematoma	01.39	434.91

Abbreviations: *ICD9-CM* international classification of diseases, ninth revision, clinical modification, *ICH* intracerebral hemorrhage, *MCA* middle cerebral artery, *TPA* tissue plasminogen activator

Additionally, the ability of ICD9-CM codes to identify patients who underwent concomitant excision of infarcted regions and who sustained hemorrhagic conversion of the infarction was evaluated among patients who underwent decompressive craniectomy for space-occupying infarction. Among the patients who underwent decompressive craniectomy for stroke, 17.4% (*n* = 8) also underwent a concomitant excision of the infarcted regions. When analyzing the accuracy of coding of the performance of excision of infarcted regions, the sensitivity of the primary procedure code was 62.5%, specificity 97.4%, positive predictive value 83.3%, negative predictive value 92.5%, and correct classification 91.3%; these values were calculated based on 5 true positive, 1 false positive, 3 false negative, and 37 true negative classifications. Moreover, among patients who underwent decompressive craniectomy, 32.6% (*n* = 15) had hemorrhagic conversion of the infarct. The ICD9-CM code (431) for intracerebral hemorrhage was evaluated to determine its accuracy for denoting hemorrhagic conversion of the infarction, and the sensitivity was 66.7%, specificity 93.5%, positive predictive value 83.3%, negative predictive value 85.3%, and correct classification 84.8%. These values were calculated based on 10 true positive, 2 false positive, 5 false negative, and 29 true negative classifications.

## Discussion

The use of ICD9-CM indicators to identify patients with acute ischemic stroke has been viewed with trepidation by some authors [17]. In 1998, Goldstein evaluated the accuracy of ICD9-CM coding, reporting a 61% accuracy for acute ischemic stroke, even when the modifier indicating infarction (which follows stenosis and occlusion of a specific artery) was considered [7]. Likewise, Reker et al. found significant variability in risk-adjusted mortality rates using ICD9-CM codes for acute ischemic stroke [9]. In a recent systematic review, McCormick et al. reported that the positive predictive value of ICD9-CM coding for acute stroke was typically less than 68% [4]; some authors, however, have found that the accuracy of ICD-CM coding of stroke has increased with time [18]. Nonetheless, patients with space-occupying cerebral infarction undergoing decompressive craniectomy represent a unique subset of patients with acute ischemic stroke [19], and the utility of ICD9-CM coding in this population remains unknown. Moreover, due to the rarity of decompressive craniectomy for stroke, single institution studies are limited by a relatively small sample size [20–28]. Thus, an effective ICD9-CM algorithm that accurately identifies patients who underwent craniectomy for stroke would allow population-based outcomes studies to be performed with greater legitimacy.

The goal of this study was to construct and validate an ICD9-CM based algorithm to identify patients undergoing decompressive craniectomy for space-occupying supratentorial cerebral infarction. This algorithm uses different ICD9-CM diagnosis and procedure codes for inclusion and exclusion. Patients were included who had one of four ICD9-CM diagnosis codes indicating acute ischemic stroke and one of five codes documenting a neurosurgical intervention; those with a diagnosis code indicating a brain tumor, subarachnoid hemorrhage, vertebrobasilar infarction, cerebral aneurysm, Moyamoya disease, intracranial venous sinus thrombosis, vertebral artery dissection, congenital anomaly of the cerebral vasculature (often used to indicate an arteriovenous malformation) [29], and head trauma, or a procedure code indicating a laminectomy (as a C1 laminectomy is a standard component of a suboccipital decompression for infratentorial stroke) [30–32] were excluded. This algorithm effectively identified patients who underwent decompressive craniectomy for supratentorial infarction, with a 97.8% sensitivity and 88.2% positive predictive value.

This complex ICD9-CM algorithm was more effective at identifying patients who underwent decompressive craniectomy for stroke than the ICD9-CM procedure code for craniectomy alone, which was only encoded in 67.4% of patients. This difference is partially because 17.4% of patients at our institution also underwent excision of infarcted territory—primarily the anterior temporal lobe—at the time of craniectomy, to reduce the risk of transtentorial herniation. Although the utility of a concomitant lobectomy (described as a strokectomy) is debated [33, 34], at our institution, its performance is determined based on the consensus of the neurosurgeon and neurocritical care team. Almost one-fifth of patients had a documented procedure code of craniotomy, however, indicating limited ability of administrative coding to differentiate between a craniectomy and craniotomy.

When the ICD9-CM algorithm was applied to admissions at our institution, there were six patients who met the criteria of our algorithm but did not undergo decompressive craniectomy for space-occupying infarction, and were therefore false positives, all of whom had an intracerebral hemorrhage. One patient had post-thrombolytic hemorrhagic conversion of cerebral infarction, another underwent decompressive craniectomy for medically refractory hypertension, while the remainder were operations for surgical evacuation of a primary intracerebral hemorrhage. The ICD9-CM indicator for intracerebral hemorrhage (431) could not be used as exclusion criteria, however, as this is the same code that represents hemorrhagic conversion of a primary cerebral infarction. Notably, another concern of using ICD9-CM indicators to identify patients undergoing craniectomy for stroke is the lack of procedure-specific ICD9 code differentiating supratentorial and infratentorial craniectomies—which represent very different operations and indications for surgery. However, the use of ICD9-CM diagnosis codes for vertebrobasilar circulation infarction and vertebral artery dissection as well as the procedure code for a laminectomy excluded all of posterior fossa craniectomies in this patient population.

Additionally, the accuracy of the ICD9 identifier 431 to denote hemorrhagic conversion of the infarction was evaluated. While the specificity of this indicator was strong (93.5%), its sensitivity was only moderate (66.7%), indicating that administrative coding of hemorrhagic conversion is less robust.

There are several limitations of the present analysis. First, as a single-center study, the proposed ICD9-CM algorithm could only be validated based on the billing codes employed at our institution. Therefore, the generalizability of this ICD9-CM based algorithm could not be evaluated with the study design, and only the internal validity could be assessed. Future analysis of the external validity of this algorithm, with confirmation of the utility of its application at other centers, will further increase the reliability of the proposed algorithm. Moreover, ICD-10 codes were not used at our institution during the years evaluated, and therefore, an ICD-10 based algorithm could not be proposed.

## Conclusions

Although the ICD9-CM code for craniectomy is not stringently coded, an algorithm of ICD9-CM diagnosis and procedure codes effectively identifies patients undergoing decompressive craniectomy for acute, space-occupying cerebral infarction. The sensitivity of the diagnosis codes to identify hemorrhagic conversion of the infarct and of the procedure codes to indicate excision of infarcted regions, however, are less robust.

## Abbreviations

ICH: Intracerebral hemorrhage
MCA: Middle cerebral artery
TPA: Tissue plasminogen activator
ICD9-CM: International Classification of Diseases, Ninth Edition, Clinical Modification
